# Comparing spatial diversification and meta-population models in the Indo-Australian Archipelago

**DOI:** 10.1098/rsos.171366

**Published:** 2018-03-07

**Authors:** Loïc Chalmandrier, Camille Albouy, Patrice Descombes, Brody Sandel, Soren Faurby, Jens-Christian Svenning, Niklaus E. Zimmermann, Loïc Pellissier

**Affiliations:** 1 Landscape Ecology, Institute of Terrestrial Ecosystems, ETH Zürich, Zurich, Switzerland; 2 Swiss Federal Research Institute WSL, 8903 Birmensdorf, Switzerland; 3 Department of Biology, Santa Clara University, 500 El Camino Real, Santa Clara, CA 95053, USA; 4 Department of Biogeography and Global Change, Museo Nacional de Ciencias Naturales, CSIC, Madrid, Spain; 5 Department of Biological and Environmental Sciences, University of Gothenburg, Box 461, SE 405 30 Gothenburg, Sweden; 6 Section for Ecoinformatics and Biodiversity, Department of Bioscience, Aarhus University, 8000 Aarhus C, Denmark; 7 Center for Biodiversity Dynamics in a Changing World (BIOCHANGE), Aarhus University, Ny Munkegade 114, Aarhus, Denmark

**Keywords:** allopatric speciation, continental drift, dispersal, diversification, meta-population model, neutral model

## Abstract

Reconstructing the processes that have shaped the emergence of biodiversity gradients is critical to understand the dynamics of diversification of life on Earth. Islands have traditionally been used as model systems to unravel the processes shaping biological diversity. MacArthur and Wilson's island biogeographic model predicts diversity to be based on dynamic interactions between colonization and extinction rates, while treating islands themselves as geologically static entities. The current spatial configuration of islands should influence meta-population dynamics, but long-term geological changes within archipelagos are also expected to have shaped island biodiversity, in part by driving diversification. Here, we compare two mechanistic models providing inferences on species richness at a biogeographic scale: a mechanistic spatial-temporal model of species diversification and a spatial meta-population model. While the meta-population model operates over a static landscape, the diversification model is driven by changes in the size and spatial configuration of islands through time. We compare the inferences of both models to floristic diversity patterns among land patches of the Indo-Australian Archipelago. Simulation results from the diversification model better matched observed diversity than a meta-population model constrained only by the contemporary landscape. The diversification model suggests that the dynamic re-positioning of islands promoting land disconnection and reconnection induced an accumulation of particularly high species diversity on Borneo, which is central within the island network. By contrast, the meta-population model predicts a higher diversity on the mainlands, which is less compatible with empirical data. Our analyses highlight that, by comparing models with contrasting assumptions, we can pinpoint the processes that are most compatible with extant biodiversity patterns.

## 1. Introduction

Understanding mechanisms of speciation and colonization throughout Earth's history is fundamental to reconstruct how life has diversified and produced biodiversity gradients [1]. Properties of species assemblages including species richness generally correlate well with contemporary ecological factors such as temperature and productivity [2], yet biodiversity has been shaped over millions of years [3]. Since the spatial distribution of ecological conditions has changed over geological time [3,4], and because those shifts have modulated speciation and extinction [5], it is important to consider palaeo-environmental conditions to understand extant biodiversity gradients [4,6,7]. Studies investigating biodiversity gradients related to current environmental patterns are almost always spatially explicit [8,9]. Moreover, spatial meta-population models [10,11] are generally implemented over ecological, not evolutionary time scales [12]. By contrast, diversification studies accounting for speciation and extinction predominantly rely on non-spatial phylogenetic analyses, and provide limited information for interpreting the processes of emergence of spatial biodiversity gradients [13,14]. Novel approaches are needed to couple reconstructions of the palaeo-environment with spatially explicit models of speciation and extinction [15,16].

Islands were recognized early as an excellent model to sharpen our understanding of the processes that shape biodiversity, including dispersal, speciation and extinction [17–19]. MacArthur & Wilson [20] formulated the theory of island biogeography that predicts species richness on islands as an emerging equilibrium between immigration and extinction, assuming rapid turnover at ecological time scales [13]. The theory of island biogeography has been extended to more complex systems [10,21], and has led to the development of meta-population models [22,23]. While these models showed some success in predicting the ecological dynamics of systems [23], they have been less effective in predicting island systems with slow colonization and extinction dynamics operating over evolutionary rather than ecological time scales [24–26]. If isolated islands are rarely colonized, immigration, speciation and extinction occur on a similar time scale as the geological changes in islands themselves (e.g. erosion) and should be considered jointly in the model [26,27]. Recent quantitative theories have successfully extended colonization–extinction processes described by MacArthur and Wilson to include speciation [26,27]. These extensions motivate the empirical evaluation of the interaction between speciation, colonization and extinction in determining species richness within archipelagos.

The processes of speciation, colonization and extinction on islands probably act at a time scale matching the dynamics of plate tectonics, island formation and geological erosion [27]. To include the geological history of islands in island biodiversity models, Whittaker *et al*. [28] proposed the immaturity-speciation pulse model of island evolution, mainly applicable to volcanic islands, where island formation and subsidence is considered to explain biodiversity dynamics [29]. These processes may not be the only ones impacting the diversity on islands over geological time scales. As continental plates move, the relative position of islands to one another has varied, affecting the rate of species colonization among them [30]. Moreover, disconnection and reconnection of islands may increase species diversity if the separation time is large enough to allow allopatric speciation. The fact that dispersal, extinction and speciation change through time in concert with the shifting configuration of islands calls for an integration of palaeo-environmental reconstructions into a theory of island biogeography. The increased availability of detailed and reliable geological reconstructions for oceanic regions [4,31–33] now provides the opportunity to evaluate whether dynamic colonization and speciation events have varied through time in relation to plate tectonic mechanisms and changing environmental conditions. Geological reconstruction can be combined to mechanistic models of speciation, dispersal and extinction [4,34] to provide a quantitative pan-biogeographic view of the evolution of biodiversity [35].

The Indo-Australian Archipelago is an outstanding centre of plant diversity [36]. For example, the floristic province Malesia alone contains approximately 42 000 vascular plant species [37]. The remarkable amount of biodiversity in this region is commonly attributed to the complexity of its geological history [35,38,39]. However, with the exception of orogeny, the direct impact of geological complexity on biodiversity dynamics has rarely been evaluated. The Southeast Asian region has undergone one of the most complex palaeo-geographical dynamics involving numerous small terranes that drifted away from Gondwana during the Palaeozoic–Mesozoic and were progressively amassed to the southern part of the Eurasian Plate at different times during the Mesozoic and Cenozoic [40]. In addition, the collision of the Eurasian and Australian plates from the Eocene–Oligocene transition onwards resulted in the creation of additional islands forming the Wallacea biogeographical region, stretching from Borneo and Sumatra to New Guinea and Australia [41]. All of these processes are expected to promote allopatric speciation through the emergence of geographical barriers to gene flow and subsequent biotic mixing when those barriers disappear and allow dispersal [35]. Yet, despite increasingly detailed tectonic models [31–33,41], the relationship between the geological dynamic of islands and plant diversity in Southeast Asia is still to be quantified using mechanistic models.

Weigelt *et al*. [42] showed how sea-level changes during glaciation can interact with the relative positions of islands and improve the explanation of island biodiversity. Alternative to correlative approaches [6,42] and theoretical perspectives [29], the use of mechanistic models allows for a quantitative evaluation of historical hypotheses and confront these with empirical data [34]. Events of dispersal and allopatric speciation can be explicitly modelled as a result of plate tectonics within the Indo-Australian Archipelago and simulations compared to observations to evaluate the compatibility with empirical data. Descombes *et al*. [34] proposed a spatial model of diversification depending on two parameters representing dispersal distances and distance thresholds beyond which gene flow is absent. Coupled with the reconstruction of the dynamics of the Indo-Australian Archipelago since the Cretaceous [33,41], it allows investigating whether simulated diversity from the model bounded by the dynamics of island patches matches observed contemporary diversity variation among islands (and adjacent continental mainland) within this archipelago. Alternatively, the current distribution of diversity might conform better to the theory of island biogeography [20], and diversity can thus be modelled as an equilibrium between current dynamics of colonization and extinction on islands. Since such an equilibrium is a larger-scale analogous to meta-population dynamics, we can model it using the meta-population model of Ovaskainen & Hanski [22]. Moreover, because the meta-population and the spatial diversification models provide outputs in the same currency (i.e. species diversity), their expectation can be directly compared.

Here, we applied both the meta-population model of Ovaskainen & Hanski [22] in its deterministic form and the diversification model of Descombes *et al*. [34] to the Indo-Australian Archipelago and confronted the model outcomes with empirical diversity data of the region. Our general aim is to compare the output of the two models that implement contrasting mechanisms and rely on different assumptions. Under the meta-population model, species diversity is expected to be higher in larger and connected patches [22], while under the diversification model, species diversity should be higher in more fragmented and dynamic parts of the island landscape [34]. We further evaluated whether the simulation results of the diversification and meta-population models matched the empirical distribution data for 14 plant families. All have diversified in this region, probably from a proto-Asian origin [34,35]. Specifically, we ask the following questions: (i) Does the diversification model that includes allopatric speciation driven by plate tectonics produce expected diversity compatible with observed diversity patterns? (ii) Alternatively, is the colonization and extinction dynamics within the meta-population model of Ovaskainen & Hanski [22] applied to extant landscape more compatible with observed diversity? Our study compares two mechanistic models, though several more have been developed recently [23,43]. Still, we expect to gain insight into the processes that have shaped biodiversity in this geographically complex region from the contrasting assumptions underlying the diversification and meta-population models.

## 2. Methods

### 2.1. Data—tropical plants in the Indo-Australian Archipelago

We assessed the match between the outputs of the two models and plant biodiversity patterns based on regional checklists of 14 fully revised families from the World Checklist of Selected Plant Families (WCSP). WCSP is an international collaborative programme providing peer reviewed and accepted scientific names of plant families and their distribution within delimited regions. In our study, we considered the following families: Apocynaceae, Araceae, Araliaceae, Arecaceae, Begoniaceae, Euphorbiaceae, Lecythidaceae, Orchidaceae, Phyllanthaceae, Podocarpaceae, Putranjivaceae, Rubiaceae, Sapotaceae and Zingiberaceae. Regional species richness (γ-diversity) of each family is provided in table 1. Those species cover many of the most diverse families in Southeast Asia and are considered representative of the diversity of the area. We used the checklists corresponding to the most detailed ‘level3’ polygons from WCSP [44]. Polygons generally correspond to countries, although larger countries are often subdivided into states, or in the case of the Indo-Australian Archipelago, islands or islands systems.

**Table 1.** Best models describing for each family current taxonomic diversity pattern. For the diversification model, we displayed the speciation and dispersal distance parameters, the mean of squared errors of the logarithm of the estimated species richness (MS.*α*), as well as the Spearman correlation value between observed and modelled species richness (cor.*α*), the mean of squared errors pairwise β-diversity (MS.*β*), the statistic of the Mantel test for pairwise β-diversity (cor.*β*). For the meta-population model, we displayed the mean of squared errors of the logarithm of the estimated species richness, as well as the Spearman correlation value between observed and modelled species richness. For all statistics, we displayed its average and standard deviation (in brackets) across resampling draws. In the last column, tests in bold indicate that the diversification model was significantly better at predicting species richness across zones, while tests in italic indicate that the meta-population model was significantly better.

		diversification model						meta-population model				comparison
family	γ-diversity	speciation (*d*_s_)	dispersal (*d*)	MS.*α*	cor. *α*	MS.*β*	cor. *β*	dispersal (log10(*d*))	log10(e/c)	MS.*α*	cor. *α*	t-test
Apocynaceae	608	15.2 (0.91)	4.0 (0)	0.377 (0.0347)	0.393 (0.0732)	0.108 (0.00791)	0.686 (0.039)	3.12 (1.47)	−4.84 (5.56)	0.563 (0.0971)	0.703 (0.0842)	***t*=**−**26 (d.f. = 260, *p* < 0.001)**
Araceae	1185	15 (4.8)	4.0 (0)	0.568 (0.0547)	0.438 (0.0721)	0.149 (0.0139)	0.636 (0.0428)	5.94 (0.435)	5.39 (2.3)	0.848 (0.141)	0.389 (0.11)	***t*=**−**26 (d.f. = 250, *p* < 0.001)**
Araliaceae	814	16.3 (6.9)	3.0 (0.069)	0.735 (0.0799)	0.355 (0.08)	0.177 (0.0179)	0.663 (0.0363)	6.04 (0.0131)	4.76 (1.85)	1.14 (0.177)	0.445 (0.0746)	***t*=**−**30 (d.f. = 290, *p* < 0.001)**
Arecaceae	1404	14 (2.8)	4.0 (0)	0.928 (0.113)	0.533 (0.064)	0.166 (0.0186)	0.68 (0.0421)	6.02 (0.247)	4.41 (1.83)	1.02 (0.15)	0.493 (0.0776)	***t*=**−**7.3 (d.f. = 370, *p* < 0.001)**
Begoniaceae	815	17.5 (1.6)	3.0 (0.12)	1.28 (0.0994)	0.591 (0.0695)	0.272 (0.0286)	0.691 (0.0405)	3.31 (0.901)	−6.18 (3.93)	2.26 (0.314)	0.603 (0.0739)	***t*=**−**43 (d.f. = 250, *p* < 0.001)**
Euphorbiaceae	1611	19 (2.5)	4.6 (0.49)	0.491 (0.0606)	0.582 (0.0496)	0.13 (0.0113)	0.614 (0.041)	5.94 (0.0167)	4.66 (1.56)	0.526 (0.0672)	0.537 (0.0657)	***t*=**−**5.6 (d.f. = 410, *p* < 0.001)**
Lecythidaceae	78	16.9 (2.9)	2.9 (0.32)	0.463 (0.0442)	0.629 (0.0563)	0.145 (0.01)	0.314 (0.0518)	2.87 (0.947)	−1.62 (1.58)	0.612 (0.0558)	0.715 (0.0734)	***t*=**−**30 (d.f. = 400, *p* < 0.001)**
Orchidaceae	11 132	17.6 (2.9)	7.0 (0.097)	0.774 (0.06)	0.399 (0.0687)	0.15 (0.0127)	0.707 (0.0331)	4.85 (1.66)	1.35 (6.34)	1.33 (0.279)	0.594 (0.171)	***t*=**−**28 (d.f. = 220, *p* < 0.001)**
Phyllanthaceae	1234	19.7 (2.2)	4.0 (0.12)	0.507 (0.0435)	0.335 (0.0723)	0.119 (0.00935)	0.69 (0.034)	5.94 (0.0112)	5.06 (1.57)	0.494 (0.0598)	0.659 (0.0522)	*t* *=* *2.5* (*d.f.* *=* *380, p* *=* *0.011*)
Podocarpaceae	125	12.9 (5.5)	3.0 (0)	0.321 (0.033)	0.769 (0.0576)	0.117 (0.0105)	0.468 (0.0612)	2.41 (0.258)	−4.02 (2.11)	0.899 (0.0983)	0.539 (0.0767)	***t*=-81 (d.f. = 260, *p* < 0.001)**
Putranjivaceae	109	8.9 (1.7)	2.3 (0.44)	0.459 (0.0625)	0.649 (0.066)	0.194 (0.0209)	0.321 (0.0529)	3.62 (1.39)	−3.57 (5.78)	0.834 (0.128)	0.538 (0.143)	***t*=**−**38 (d.f. = 300, *p* < 0.001)**
Rubiaceae	5250	18.2 (2)	6.0 (0.069)	0.755 (0.0824)	0.225 (0.0789)	0.159 (0.015)	0.724 (0.0332)	6.01 (0.0102)	4.54 (1.69)	0.747 (0.0949)	0.542 (0.0603)	*t* = 0.95 (d.f. = 410, *p* = 0.34)
Sapotaceae	528	10.1 (2.6)	3.0 (0.069)	0.495 (0.0381)	0.675 (0.0474)	0.161 (0.016)	0.367 (0.0468)	5.7 (0.881)	2.44 (1.5)	1.03 (0.139)	0.405 (0.12)	***t*=**−**53 (d.f. = 240, *p* < 0.001)**
Zingiberaceae	1435	16 (0)	4.0 (0)	1.2 (0.118)	0.362 (0.0841)	0.215 (0.0204)	0.678 (0.0416)	4.56 (1.68)	−1.08 (7.13)	1.89 (0.384)	0.565 (0.196)	***t*=**−**25 (d.f. = 250, *p* < 0.001)**

### 2.2. Biodiversity models

#### 2.2.1. An historical perspective: the diversification model

We used a model of spatial diversification through time [4,34] on reconstructed palaeo-habitats in the Indo-Australian Archipelago region. The model uses a reconstruction of terrestrial palaeo-habitats depicting the evolution of continental blocks, ocean basins and land distributions at 0.5° resolution for the past 140 Ma, with a temporal resolution of 500 kyr time steps [33]. Original maps were converted into grids of 0.5° resolution for each time step representing oceans and putative land surfaces and by keeping the original spatial projections. Because climate has strongly fluctuated during this period [45] and our study primarily focuses on tropical plant families, we reconstructed the palaeo-latitudes of the tropical border as an additional habitat constraint. We used reef-forming coral fossil records, which are good indicators of tropical climates since they mostly develop in water temperature greater than 25°C. We collected all reef-forming coral fossil occurrences from the Fossilworks database [46] and reconstructed the tropical border latitude for each 0.5 Ma step on an equal area grid at a resolution of 0.5° using the 95th percentile of palaeo-latitudinal limits. As older fossils became scarcer and present a higher dating uncertainty, we considered a dating uncertainty of 3 Ma for fossils younger than 10 Ma, 5 Ma for fossils between 10 and 50 Ma and 10 Ma for fossils older than 50 Ma.

Following Gotelli *et al*. [16], the diversification model keeps track of the distribution of each species in each cell of a grid of suitable habitats (here landmasses) as well as phylogenetic relationships between species at any single point in time. The core of the model is based on the assumption that species can disperse and speciate into sister species as a consequence of the shift in habitat configuration [4,34]. The Indo-Australian Archipelago is assumed to be a closed system, and the diversification starts from the simplifying assumption of a single species present in Asia 140 Ma ago. For each time step of the diversification model, three phases are distinguished: (i) speciation phase: allopatric speciation arises when a species range is split into one or more distinct areas separated by a minimal sea distance *d*_s_; (ii) dispersal phase: at each time step, all species disperse according to an identical dispersal parameter; species in the time step *t* are allowed to disperse to all habitat cells at the time step *t* *+* 1 that are distant by a value lower than *d*; dispersal was modelled by a Weibull distribution (shape = 1) assuming more frequent events at short- compared to long-distance dispersal and dispersal values were drawn from the kernel for each species at each time step; (iii) extinction phase: if all habitat cells inhabited by the species at time *t* disappear at time *t* *+* 1 and no other habitat cells at dispersal distance lower than the dispersal threshold *d* emerges at time *t* *+* 1, the species gets extinct [34]. Hence, extinction is not stochastic and only occurs when the emerged land on which a species is distributed disappears without replacement. A detailed explanation of the model architecture is available in Descombes *et al*. [34].

We started the simulations with one species occupying a continuous range of available habitat in the most ancient time period in the Asian palaeo-continent at 140 Ma, as the families considered were assumed to be of proto-Asian origin. We ran the simulations for a range of dispersal (*d* ∈ {0.5°:20°}) and speciation (*d*_s_ ∈ {0.5°:20°}) distances. The minimum distance corresponds to the cell size used, while the maximum distance is an extremely long dispersal distance that is very rare among plants. Hence, we explored the full realistic range of dispersal and speciation distance parameters. Moreover, we only explored simulations in which the dispersal parameter *d* is lower than speciation parameter *d*_s_. Values beyond this range produced unrealistic species numbers (i.e. much higher than the maximum of the richest family, i.e. approx. 11 000 for the Orchidaceae).

#### 2.2.2. A meta-population perspective: colonization and extinction across Southeast Asia

To model the colonization and extinction within the current spatial arrangement of land patches, we used the patch dynamic model of Ovaskainen & Hanski [22] in its deterministic form across the most recent map of land. The model was run over the same area as the diversification model, consisting of the tropical zone of Southeast Asia. Similar to the diversification model, each patch *i* of area *A_i_* is considered to be linked to others through a cost-distance *d_ij_*. The probability of a colonization event for a species from one patch to another is determined by the mean dispersal distance *d.* The vector of probability *P* = {*p_i_*} of a patch to be occupied by a given species can be written as follows:

$$\frac{\text{d}p_{i}}{\text{d}t}=c\times \left[\underset{j\neq i}{\sum }p_{j}A_{j}{\text{e}}^{-d_{ij}/d}\right]\times (1-p_{i})-\frac{e}{A_{i}}p_{i}$$

where *c* and *e* were, respectively, the colonization and extinction rates, *d_ij_* the cost-distance between patches *i* and *j,* and *d* the average dispersal distance (note that this dispersal parameter is not related to the dispersal parameter *d* of the diversification model). Colonization was implemented such that colonization probability decreased exponentially with distance. We ran the model over the set of patches defined by the WCSP zones within the tropical boundaries consisting of a network of 26 patches, whose areas were computed as the sum of their constituting cells. We considered that two patches were separated by a dispersal distance equal to the minimal distance between their cells. We used an ODE23 engine to run the probability estimation of species occupancy within each cell. We estimated the vector *P* for a range of dispersal (log10(*d*) ∈ {0,6.5}) and the ratio of colonization and extinction rate (log10(e/c) ∈ {5.4,5.8}). Given a set of *N* species in the region (γ-diversity), we estimated the number of species in each patch *Np_i_*. In complement to the meta-community model at the resolution of the WCSP zones, we ran the meta-population model at the finer resolution of the cell.

### 2.3. Comparison of model outputs to plant diversity data

#### 2.3.1. Simulation of taxonomic α-diversity from the diversification model

We compared the contemporary species richness for each of the 14 tropical plant families with the simulation results of the diversification model. We upscaled the resolution of the cell-based simulations from the diversification model to fit the regional resolution of the plant occurrence data, from 5580 cells to 26 zones, by aggregating species lists of all cells within a region. We then compared results for the 26 zones that were entirely comprised within the study area. Species richness values were log-transformed to avoid giving disproportionate weight to the richer zones. We retained the parameter set of the models that returned the lowest mean square errors across zones using a jackknife resampling scheme. We sequentially parametrized the model on 24 zones and projected the model to the remaining two zones. We used the collated vector of independently projected diversity to compute the mean square error and estimated the reliability of the speciation and dispersal distance values. While this does not provide a fully independent evaluation of the models as is normally recommended (e.g. [47]), it is the best available approach given that an independent dataset is unavailable. We further reported the following performance indicators: Spearman rank correlation among observed and modelled richness, error rate in β-diversity as well as the Spearman rank correlation among observed and modelled β-diversity (using a Mantel test). Dissimilarity among regions was expressed by the pairwise Sorensen dissimilarity index, which is a linear transformation of a metric of β-diversity *sensu stricto* [48]. These indicators were also estimated on the training set of zones across resampling draws but were not used to select the best parametrization.

#### 2.3.2. Simulation of taxonomic α-diversity from the meta-population model

For each family, we compared predicted and observed values of species richness using the mean-squared errors and retained the parameter set of the models with the lowest value. To avoid overfitting, we used the same resampling scheme as shown above. We sequentially parametrized the model on 24 zones and projected the model on the remaining two zones to compute independent performance statistics. We reported for each family the best parameter values computed from this semi-independent evaluation. We further reported the following performance indicators: error rate in richness as well as the Spearman rank correlation among observed and modelled richness. These values were estimated on the training set across resampling draws. For each family, we then compared the distribution of mean-squared errors in α-diversity for the best parametrization between the two models using a *t*-test and assessed if the two models return significantly different error rates.

#### 2.3.3. Comparisons of observed and modelled phylogenies

For Arecaceae, we further investigated whether the simulations from the diversification model were capable of predicting the shape of the regional phylogeny. We used a species-level phylogeny of Arecaceae [49], and compared its shape to the phylogenies predicted by the diversification model using three metrics: the number of species, the Colless index that measures imbalance compared to the hypothesis of a Yule ‘pure birth’ process [50], and Gamma values which quantify the ‘tippiness’ versus ‘stemminess’ of a phylogenetic tree. A tippy phylogeny shows longer inter-nodal distances towards the tips, and vice versa. Using these statistics, we compared the Arecaceae phylogeny to the simulated phylogeny from our diversification model for the best parametrization estimated as described above.

### 2.4. Regional scale α- and β-diversity analyses through time

From the simulations showing the best fit to extant biodiversity patterns, we evaluated the biodiversity dynamics within the Indo-Australian Archipelago during the last 140 Ma using a decomposition of α-, β- and γ-diversity. At each time step, we computed the mean α-diversity as the average number of species present in an occupied cell and the β-diversity as the ratio between the total number (*γ*) of species present at this time step across all occupied cells and the mean α-diversity. This formulation of the β-diversity has the advantage of returning a landscape-scale estimate of β-diversity that is independent of α-diversity [49]. We related α- and β-diversity to time in order to determine how major plate tectonic movements shaped the modelled regional diversity dynamics. All analyses except the meta-population model simulations were done using R3.4.3. Meta-population model simulations were computed using the software MATLAB R2015a.

## 3. Results

### 3.1. Overview of the output of the two dynamic models

The two models generally returned contrasting patterns of present diversity. Nevertheless, each of the models produced consistent spatial diversity patterns across the explored parameter space (electronic supplementary material, figure S2). The diversification model predicted that the Southeast Asia Archipelago is a hotspot of diversity with more species than continental Southeast Asia and Australia, while the meta-population model predicted the inverse pattern, with the Archipelago having a lower diversity than the continents (electronic supplementary material, figure S4).

### 3.2. Comparisons of taxonomic α-diversity

#### 3.2.1. Diversification model

**Figure 1.**
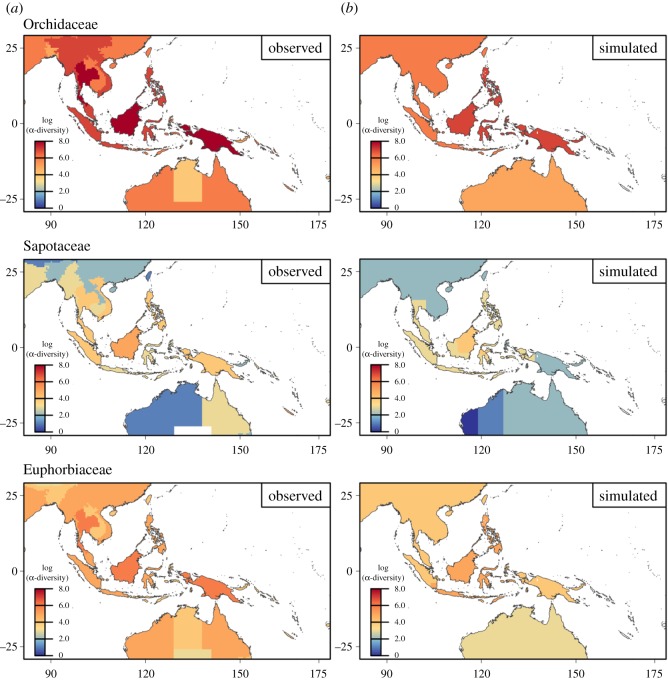
Observed species richness for three selected plant families among the 14 studied. The first column (*a*) represents the observed species richness for the Orchidaceae, Sapotaceae and Euphorbiaceae in the Southeast Asia Archipelago. The second column (*b*) represents the best predicting simulation drawn from the diversification model for each one of them for the following parametrization: *d*_s_ = 18 and *d* = 7, *d*_s_ = 10 and *d* = 3, *d*_s_ = 19 and *d* = 5, respectively.

**Figure 2.**
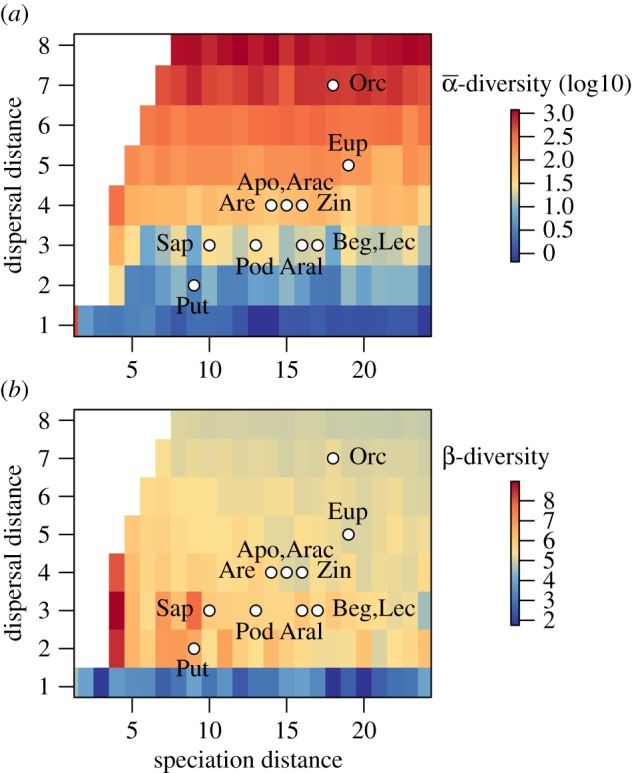
Modelled best speciation and dispersal distances for each family. Families names are abbreviated as follow Apo: Apocynaceae; Arac: Araceae; Aral: Araliaceae; Are: Arecaceae; Beg: Begoniaceae; Eup: Euphorbiaceae; Lec: Lecythidaceae; Orc: Orchidaceae; Pod: Podocarpaceae; Put: Putranjivaceae; Sap: Sapotaceae; Zin: Zingiberaceae. This parametrization reflects the averaged best parametrization of modelled with observed diversity of each family based on species richness. Phyllanthaceae and Rubiaceae were not represented as their richness pattern is best predicted by the meta-population model. The background colours represent in (*a*) the variability of the α-diversity and in (*b*) variability of the β-diversity among simulations. The white background colour delimits the unexplored parameter space.

**Figure 3.**
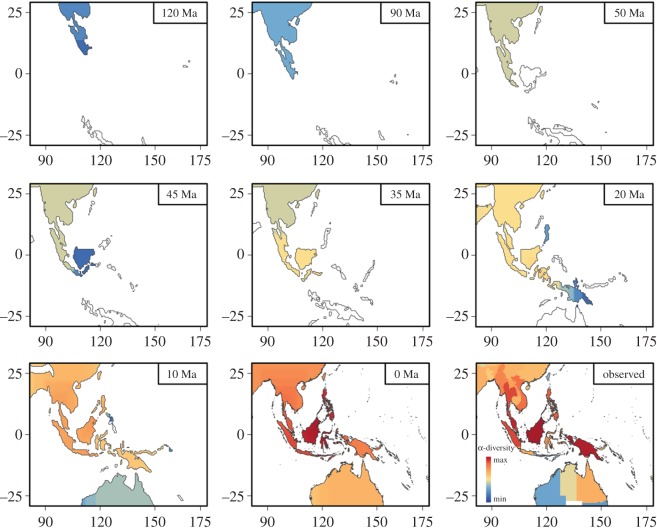
Map of estimated total species richness through time upon the layers of the continent position, based on the diversification modelling (*d*_s_ = 14, *d* = 4). The final map of the panel shows the total observed species richness. The species richness data are log transformed to better illustrate the pattern of diversity through time.

There was substantial variation in the parameter estimates across families, and in the performance of the best model for each family (table 1). Estimated dispersal parameters showed a low standard deviation, while estimation of speciation distances were generally more variable, in particular for the families Araceae, Araliaceae and Podocarpaceae. For α-diversity, the correlation between the prediction from the best model and the observed data ranged from 0.23 (Rubiaceae) to 0.77 (Podocarpaceae, figure 1). The correlation between modelled and observed α-diversity among the 26 zones was significant (*p* < 0.05) for 7 out of 14 families and marginally significant (0.05 < *p *< 0.1) for two of them (table 1). The observed β-diversity showed significant correlation with modelled β-diversity in all families ranging from 0.31 (Lecythidaceae, table 1) to 0.71 (Orchidaceae, *p* < 0.05, table 1). Simulations with most parameter sets predicted distinct extant distribution hotspots of species richness, with peaks in Borneo, New Guinea, The Philippines and, to a smaller extent, in Eastern China, which correspond well with observed species richness gradients in most of the families (figure 1 and electronic supplementary material, figure S1). The best-fitting simulations revealed different parameter sets for the different families (figure 2). We found a general discrepancy between modelled and observed diversity for several families for the island of Sulawesi, which was predicted to have a high species richness, but shows low observed diversity in all families (figure 3).

#### 3.2.2. Meta-population model

Overall, the meta-population model returned higher errors (MS.*α*, table 1) than the diversification model for all clades except for the Rubiaceae and the Phyllantaceae. In the last case, the meta-population model was significantly better in predicting richness pattern across zones. Furthermore, for 7 of the 14 families, the estimation of the parameters did not stabilize (high standard deviation). While the errors were generally higher, the meta-population model predicted the hierarchy of the WCSP regions with higher accuracy than the diversification model for half of the families (table 1), which principally display a large diversity on continental Asia (electronic supplementary material, figure S1): Apocynaceae, Araliaceae, Begoniaceae, Lecythidaceae, Orchidaceae, Phyllanthaceae, Rubiaceae and Zingiberaceae.

### 3.3. Comparisons of phylogenies

Regardless of the parametrization, the diversification model predicted that the phylogeny should be more balanced than that predicted by a Yule ‘pure-birth’ process (Colless tests were not significant for 95% of the scenarios and standardized Colless index varied between −1.45 and 7.02). The diversification model produced tippy phylogenies (93% of the scenarios returned a positive *γ* statistic that varied between −13.4 and 48.0), consistent with an increasing rate of lineage accumulation. While the observed Arecaceae phylogeny was as tippy as modelled phylogenies (*γ* = 13.62) and also not significantly different from a Yule ‘pure-birth’ process, the standardized Colless statistic (6.88, *p* = 1. 00) was high compared to modelled phylogenies indicating that it was more imbalanced in regard of a Yule ‘pure-birth’ process (electronic supplementary material, figure S3).

### 3.4. Spatial biodiversity dynamics

**Figure 4.**
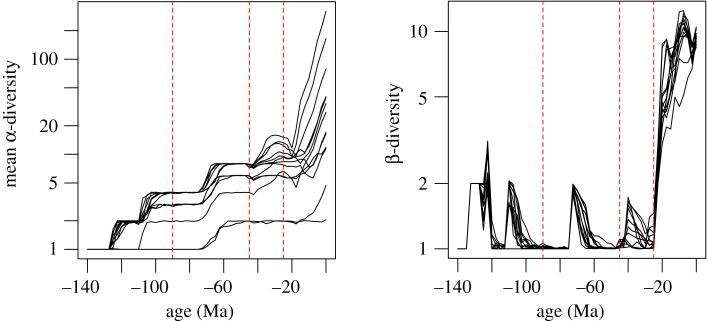
Dynamics of the simulated mean α-diversity and β-diversity across cells for the parametrizations that best fit the simulated with observed data (cf. table 1). Dates highlighted in red correspond to: the apparition of an island that prefigures Borneo (90 Ma), the apparition of islands that will become part of The Philippines islands (45 Ma) and the meeting of the northern and southern archipelagos (25 Ma).

Throughout all simulations, biodiversity was shaped by periods of major exchange among the different land patches. While central islands such as Borneo accumulated biodiversity rapidly, more isolated or peripheral land patches such as Java, Bali or the islands of Andaman did not accumulate as much biodiversity (figure 4). The simulations with the different parameter sets optimized for each family showed consistent patterns of diversity dynamics through time, with a gradual accumulation of α-diversity and strong fluctuations of the mean β-diversity within the Indo-Australian Archipelago (figure 4). Several events of colonization and disconnection promoted a few peaks in β-diversity, but consecutive reconnections led to a drop in β-diversity. According to the simulations, a steadier increase in β-diversity was only achieved after 50 Ma, when the complexity of the archipelago increased and a colonization of Australia through New Guinea became possible (figure 4).

## 4. Discussion

Plate tectonics is considered a major force shaping biodiversity [4,51,52]. In his pan-biogeographic synthesis of biogeography together with geology, Heads [35,53] emphasized the role of plate tectonics in fostering isolation and speciation in the Australasian region. In geologically complex regions, the spatio-temporal dynamics of land patches should modulate species opportunities for dispersal, speciation and also cause extinction, when habitats are lost [34,35]. Our results show that a diversification model bounded by the dynamics of the configuration of land patches in the Indo-Australian Archipelago generates richness patterns that are in agreement with empirical patterns. Across a wide range of parameters, the model systematically predicted a higher diversity on Borneo, which has a central position within the network of island patches. The species diversification model performed generally better than a classic meta-population model, suggesting that beyond contemporary species colonization-extinction dynamics, a historical and spatial signal of speciation exists in the distribution of plant species diversity within the Indo-Australian Archipelago. Both models remain conceptually simple and in the future species diversification should be integrated with meta-population and niche models towards a more realistic picture of biodiversity in dynamic landscapes [34].

The diversification and the meta-population models both provide output at the same currency, i.e. species richness per patch, though based on very different assumptions. The meta-population model inspired by the theory of island biogeography [20,22] expects that larger donor patches sustain higher diversity than more isolated and smaller ones [22]. By contrast, the diversification model predicts a larger diversity within more fragmented parts of the landscape, where allopatric speciation is more likely to occur [4,34]. Despite differences in underlying assumptions, the two models are comparable in several aspects. They display similar levels of complexity and are explored across two parameter axes: dispersal and speciation distance for the diversification model [34]; dispersal distance and ratio between colonization and extinction rate for the meta-population model [22]. Moreover, they both consider species as independent without the effect of biotic interactions [34]. When compared to data, the diversification model showed a higher agreement with observed species richness than the meta-population model (lower MS.*α*). Hence, the model relying its dynamic on plate tectonics better represents the diversity pattern of the Indo-Australian Archipelago. The model generally predicts a higher diversity in the archipelago, especially Borneo, compared to mainland locations, congruent with empirical data (electronic supplementary material, figure S1). By contrast, according to the meta-population model, the Asian and Australian mainlands act as biodiversity reservoirs, with higher diversity, while the archipelago acts as a biodiversity sink. The higher match of the diversification model with empirical data agrees with the hypothesis that geological dynamics associated with species dispersal, extinction and speciation have generated the major spatial biodiversity gradients in the Indo-Australian Archipelago.

While the theory of island biogeography underlying the meta-population model emerged several decades ago [20], the development of the diversification model stems from a recent initiative to account for mechanisms and palaeo-environmental changes to explain biodiversity gradients [4,16,43]. A link between geology and biogeography was already postulated earlier [35], but was rarely tested within a quantitative framework. Previous attempts have considered habitat dynamics in spatial diversification models. For instance, Jordan *et al.* quantified the role of plate tectonics in shaping terrestrial diversity using an individual-based model [54]. By contrast to individual-centred approaches [55,56], the diversification model considers species ranges as the modelling unit, which makes it much faster to process many temporal environmental maps with large spatial extents and coarser resolutions [4,34]. An advantage of the diversification model is to provide a variety of outputs, such as the expected shape of the phylogeny, which can be compared to empirical phylogenies. In the current study, the comparison of the phylogeny of Arecaceae did not provide further support of the diversification model. The model predicted a very specific phylogenetic tree shape with a balanced phylogeny and nodes concentrated towards the tips. This specific structure was not congruent with the phylogenetic composition of palm lineages. This suggests that other processes such as trait evolution and local niche processes should be included in future models to allow for predicting more realistic phylogenetic tree shapes [57]. While the diversification model generates results for many different properties, so far only species diversity has been compared. In order to more convincingly demonstrate the legacy of geological dynamics, other properties should be explored based on phylogenies or from the fossil record [34].

Results from the diversification model are consistent with the idea that land connections followed by protracted isolation have primarily contributed to shape the biodiversity patterns in the Indo-Australian Archipelago. The historical floristic patterns of the Indo-Australian Archipelago have been previously associated with plate tectonics based on the fossil records [35]. Tectonic changes have been hypothesized to separate more widespread insular meta-populations and to produce endemics restricted to fewer or even single islands [58], similar to the process modelled in our study. The diversification model suggests that the shifting positions of landmasses relative to each other, especially in the archipelago, have constituted a biodiversity pump as efficient as orogeny. Allopatry is obviously a powerful evolutionary model to explain biodiversity gradients in the Indo-Australian Archipelago [35]. Our model correctly predicts the empirical biodiversity gradients characterized by a diversity peak in Borneo (figure 1). The continental island of Borneo has a singular geometric position at the centre of a star-like network connecting the Asian continent, the Malaysian Peninsula, The Philippines and Indonesia. This central position in the network of land patches coupled with the transient nature of connections seems to have enhanced biodiversity accumulation. Our results agree with previous simulations showing how geometric constraints can shape diversity in the mid-domain of a bounded area [59].

The diversification model thus provides a likely scenario for the emergence of biodiversity in the Indo-Australian Archipelago. According to our model, New Guinea was within dispersal reach from Sulawesi and indirectly from Australia at regular time intervals, which allowed species exchange followed by periods of isolation with consecutive speciation events. The emergence of biodiversity followed as a consequence of the geological dynamics. The temporal landscape α- and β-diversity from simulations fitting extant data revealed an acceleration in the Oligocene to Miocene, when the geographic configuration of the archipelago increased in complexity, and a connection may have appeared from Asia to New Guinea [39]. Our simulation results agree with pollen records, which suggest that the main exchanges between the Asian and Australian plates occurred as early as the Miocene and continued to shape current assemblage richness [60]. We found that the pattern of accumulation of α-diversity largely differed from β-diversity through time. While the number of species accumulated regularly across the region, the β-diversity patterns showed strong fluctuations, which illustrate the influence of plate tectonics as a speciation pump. Periods of isolation generated endemic species diversities within each land patch, enhancing β-diversity, but subsequent reconnections allowed for exchange among land patches [61,62] and a decline in β-diversity. By contrast, species mean α-diversity continued to accumulate across the region throughout these successive exchanges. The flora of the Indo-Australian Archipelago was simulated to have become probably enriched during successive sequences of widespread migration from Asia, through the Malay Peninsula and Borneo.

The speciation and dispersal distance parameters fitting empirical data varied considerably among the families considered. Orchidaceae had comparatively larger dispersal values and exhibited larger species richness beyond the Wallace line. According to our model results, the comparatively larger diversity in New Guinea arose from more frequent dispersal events from Wallacea forming new species. Orchidaceae have extremely light seeds [63], and are expected to disperse the farthest, possibly in interaction with animals [64], which is in agreement with the model results. The model generally underestimated the α-diversity in New Guinea, especially for the Orchidaceae. This region is characterized by a complex topography with the highest peak reaching up to 4509 m of elevation. Hence, speciation in this island might have been driven also by orogeny as found in other mountain ranges [51,65] and not only by pure plate movements as modelled here. The model also tends to overestimate the species richness in Sulawesi. The rainforest ecosystems in that region suffered several episodes of drought during the Quaternary [66], which might have caused extensive species extinctions. Future diversification models based on plate tectonics would benefit from including speciation from orogeny, and the Quaternary climate to explain archipelago biodiversity dynamics.

The Indo-Australian Archipelago has a large number of endemic groups and disjunctions as well as a complex tectonic background [35]. Similar patterns of the distribution of diversity can be found for numerous unrelated groups underlying that a similar process is shaping diversity [35,58] (electronic supplementary material, figure S1), possibly related to the geological history of this region. Developments in mechanistic models [34,67] and geological reconstruction [41] allow for quantitative pan-biogeographic analyses. The results of the spatial diversification model suggest that isolation of populations through the creation of oceanic channels fuelled by dispersal during periods of reconnection is the mechanism by which plate tectonics may have acted as a major catalyst of allopatric speciation. Expectations from the diversification model are more compatible with empirical data than the meta-population model assuming a stable landscape. Hence, despite the current limitations of the diversification model (i.e. no topography, no effect of the Quaternary glaciations), it is still a very useful method to illustrate that the dynamics in the geographic configuration of islands is sufficient to reproduce patterns like those of species diversity. Our study adds evidence to the idea that geological dynamics are a major force in building biodiversity [4,68], complementing historical and current climate in shaping extant assemblages. Within the context of island biogeography, the Indo-Australian Archipelago is a special case with many continental islands. The diversification and meta-population models should be further explored over oceanic islands with shorter life spans to draw more general conclusions [69].

## Supplementary Material

Figures S1 - S4

## Data accessibility

The data supporting this article are available on the Kew online database http://wcsp.science.kew.org/home.do and from the Figshare link https://doi.org/10.6084/m9.figshare.5831319.v1.
