# Supplementary material for: Comparing spatial diversification and meta-population models in the Indo-Australian Archipelago
Source: R Soc Open Sci. 2018 Mar 7;5(3):171366. doi: 10.1098/rsos.171366 (PMC5882677; doi:10.1098/rsos.171366)

**Supplementary information**

**Plate tectonics and floristic diversity in the Southeast Asian archipelago**

Loïc Chalmandrier, Camille Albouy, Patrice Descombes, Brody Sandel, Soren Faurby, Jens-Christian Svenning, Niklaus E. Zimmermann, Loïc Pellissier

Figure S1: Observed species richness for the fourteen studied plant families in the Indo-Australian Archipelago based on the Kew species lists.


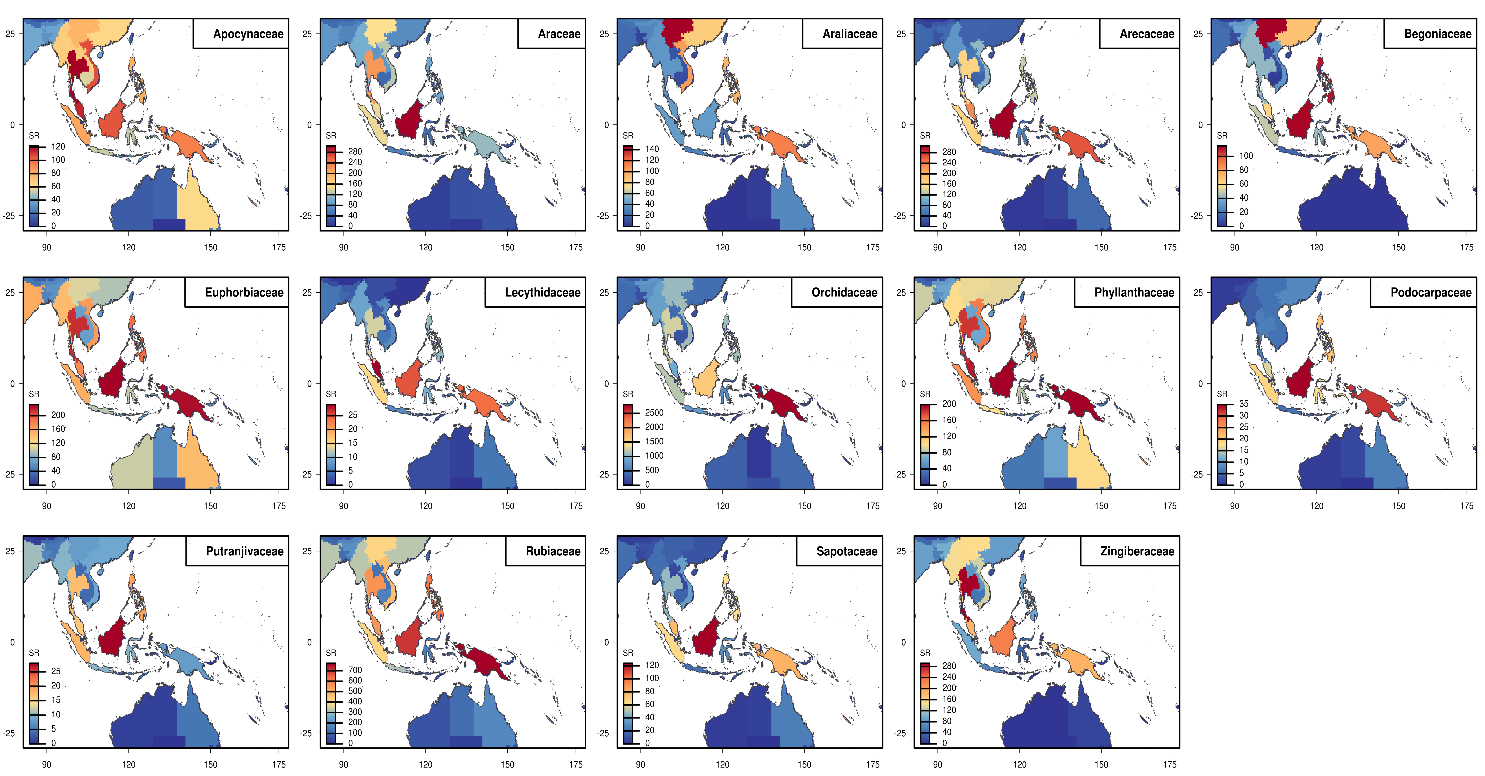


Figure S2: Predicted *α*-diversity according to the simulation best matching observed plant diversity patterns. The set of speciation (*d_s_*) and dispersal (*d*) distance parameters are indicated for each simulation.


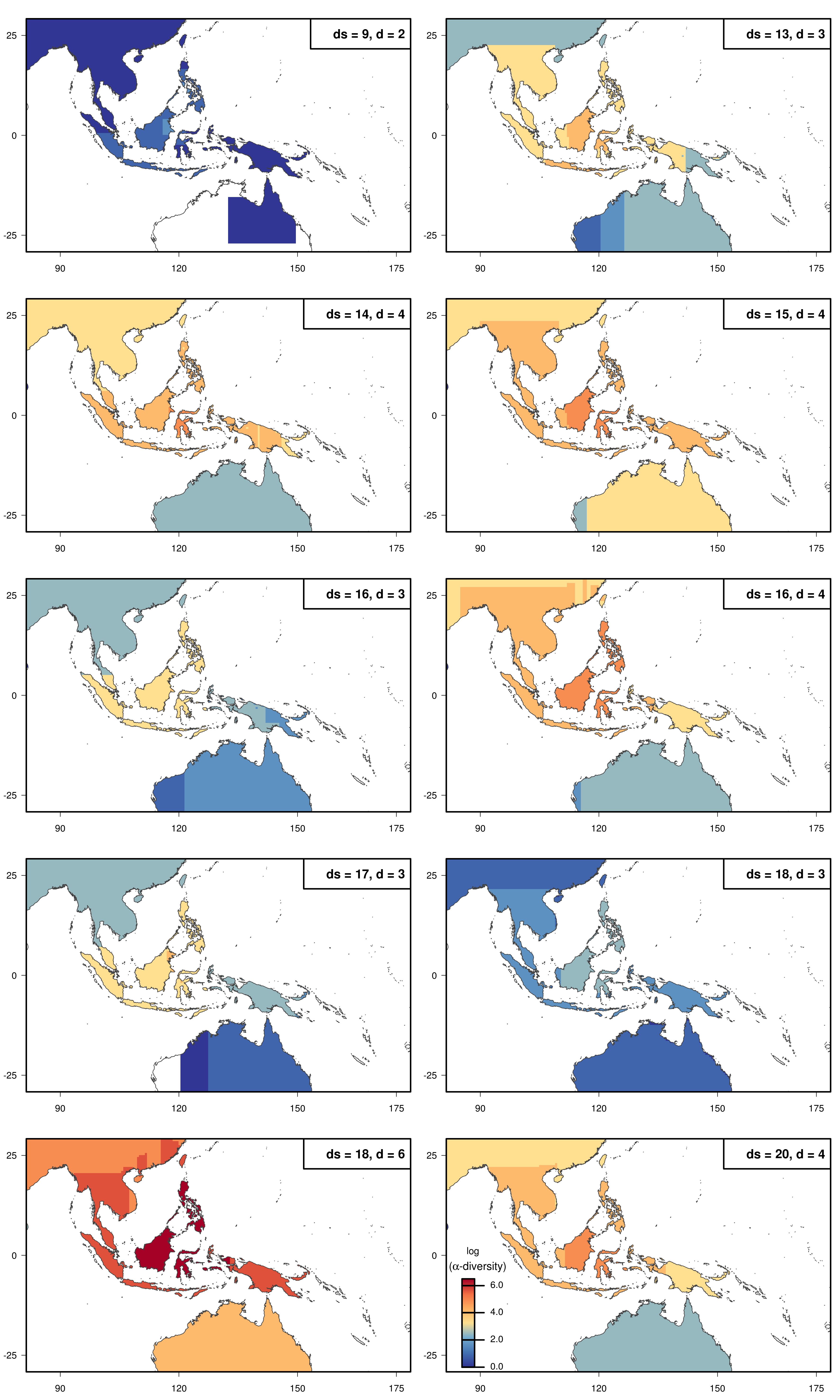


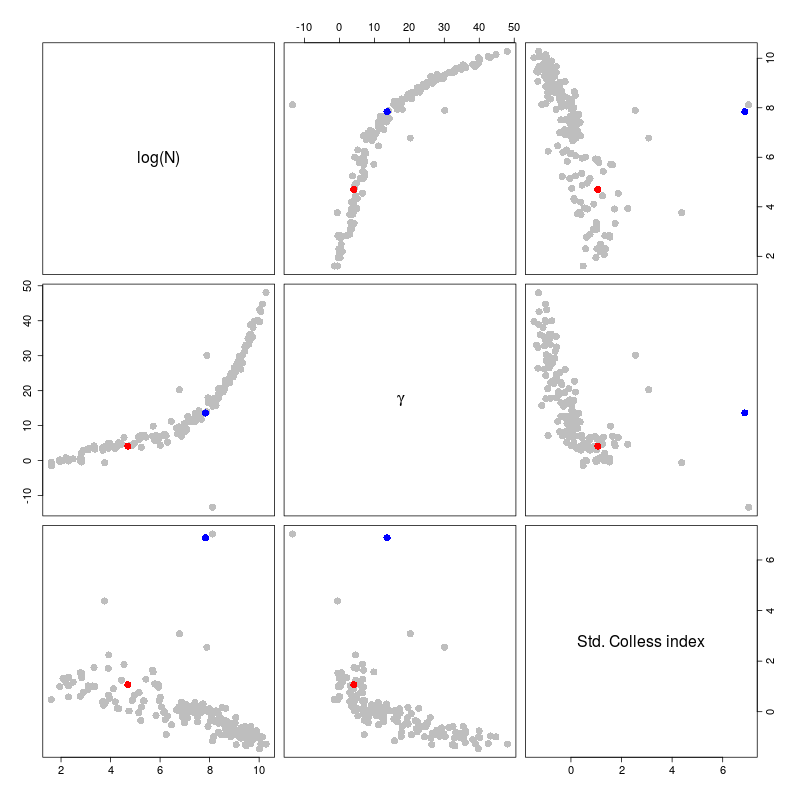
Figure S3 – Phylogeny shape characteristics as predicted by the diversification model: *N*, number of species ; *γ* indicates if nodes are concentrated towards the root (negative values) or the tips (positive values) ; and the Colless index describes if the phylogeny is more balanced (positive values) than predicted by the hypothesis of a Yule “pure birth” process. In blue, is indicated the values describing the empirical phylogeny of the Arecaceae and in red the characteristics of the phylogeny from the “best” simulations (*d_s_* = 12, *d* = 3).

Figure S4 – In complement to the meta-community model at the resolution of WCSPF zones, we run the meta-population model at the finer resolution of the cell. We used an ODE23 engine to run the estimation of probability of occupancy of each pixel. We used the software MATLAB (MATLAB R2015a. - The MathWorks, Inc.). One limitation of this higher resolution run of the meta-population model is that the estimation does not keep track of the among-patches occupancy probabilities. Consequently, we cannot use this model at the pixel scale to fit the diversity pattern observed at the scale of the WCSPF zones and we cannot use it to estimate β-diversities between zones. Nevertheless, the meta-population model can be compared to the diversification model at the pixel resolution. We estimated the vector P for a range of dispersal (d’ ∈ {0.5°:20°}) and ratio of colonisation and extinction rate (log10(c/e) ∈ {X;Y}). Given a set of N species in the region (γ-diversity), the estimated number of species in each zone is the pixel i is Npi. We compared the outputs of the diversification model and the fine scale meta-population model by calculating the mean pixel diversity in three different subregions: the Asian mainland, South East Asian Archipelago and Australia and analyzing the species richness hierarchy between the three regions. Difference in average species richness between subregions of the South-East Asian Archipelago as predicted by the diversification model (left) and the meta-population model (right). We compared the difference between the Archipelago (Arc. ), Asian mainland (Asia) and Australia mainland (Aus. ). Comparing the models at the resolution of pixel, the geological and metapopulation model predicts different diversity hierarchy between the three main areas of the region: Asian mainland, Archipelago and Australian mainland. The diversification model predicts that locations situated in the Archipelago should be richer than locations on the Asian and Australian mainlands (Figure S4). The pattern was consistent across parametrization of dispersal and speciation distance, except for low values of dispersal distance (inferior to 2) where locations on the Asian continent were richer than the others. Contrasting with this outcome, the meta-population model systematically predicts a higher diversity in the locations of the Asian and Australian continent compared to the Archipelago (Figure S4).


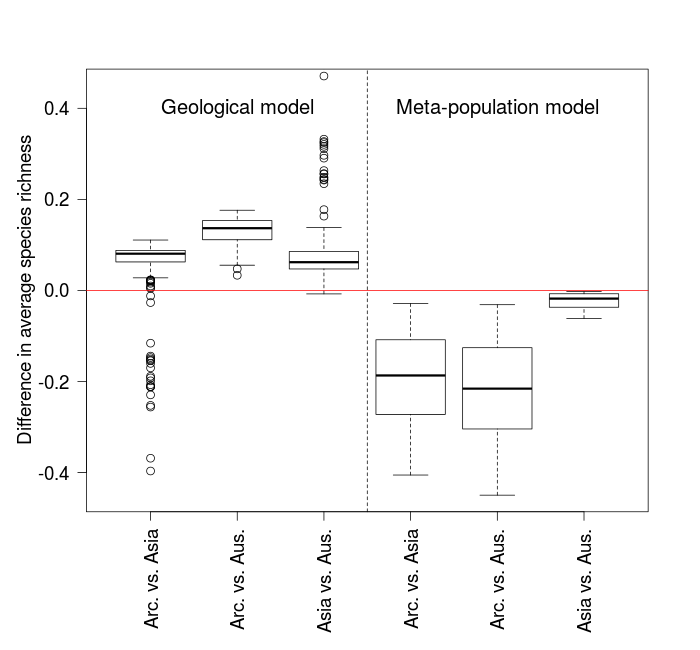

Supplement: Figures S1 - S4 [file rsos171366supp1.docx]
